# Decision theory for precision therapy of breast cancer

**DOI:** 10.1038/s41598-021-82418-7

**Published:** 2021-02-19

**Authors:** Michael Kenn, Dan Cacsire Castillo-Tong, Christian F. Singer, Rudolf Karch, Michael Cibena, Heinz Koelbl, Wolfgang Schreiner

**Affiliations:** 1 Section of Biosimulation and Bioinformatics, Center for Medical Statistics, Informatics and Intelligent Systems (CeMSIIS), Medical University of Vienna, Spitalgasse 23, 1090 Vienna, Austria; 2 Translational Gynecology Group, Department of Obstetrics and Gynecology, Comprehensive Cancer Center, Medical University of Vienna, Waehringer Guertel 18-20, 1090 Vienna, Austria; 3 Department of General Gynecology and Gynecologic Oncology, Medical University of Vienna, Waehringer Guertel 18-20, 1090 Vienna, Austria

**Keywords:** Breast cancer, Gene expression, Diagnostic markers, Applied mathematics

## Abstract

Correctly estimating the hormone receptor status for estrogen (ER) and progesterone (PGR) is crucial for precision therapy of breast cancer. It is known that conventional diagnostics (immunohistochemistry, IHC) yields a significant rate of wrongly diagnosed receptor status. Here we demonstrate how Dempster Shafer decision Theory (DST) enhances diagnostic precision by adding information from gene expression. We downloaded data of 3753 breast cancer patients from Gene Expression Omnibus. Information from IHC and gene expression was fused according to DST, and the clinical criterion for receptor positivity was re-modelled along DST. Receptor status predicted according to DST was compared with conventional assessment via IHC and gene-expression, and deviations were flagged as questionable. The survival of questionable cases turned out significantly worse (Kaplan Meier *p* < 1%) than for patients with receptor status confirmed by DST, indicating a substantial enhancement of diagnostic precision via DST. This study is not only relevant for precision medicine but also paves the way for introducing decision theory into OMICS data science.

## Introduction

### Background and significance

Precision medicine relies on biomarkers for selecting the most adequate therapy for each single patient^1–3^. In particular, individually optimized treatment of breast cancer patients has widely been studied, drawing on molecular subtyping^4–7^, networks and pathways^8–12^ or more general gene expression signatures^13–20^. Among such signatures, PAM50^21,22^, PREDICT^23^, the Gene expression Grade Index^24^ and the Genomic Grade Index^25^ have been widely recognized. For a survey see Huang, C. C. et al.^26^.

Hormone receptor status (estrogen, ER and progesterone, PGR) as well as the human epidermal growth factor 2 receptor (HER2) are considered most important^27,28^ and are estimated by immunohistochemistry (IHC) in clinical practice^29–36^. If at least one of both receptors (ER or PGR) is found positive (i.e. receptors are present), hormone therapy will suffice for an effective treatment, avoiding the much more severe side effects of chemotherapy. However, if the receptor status should accidentally be estimated falsely positive, hormone treatment will not work and the patient may be deprived from life-saving chemotherapy.

Due to its focal importance, receptor assessment quality has been scrutinized in multicenter studies^37,38^, suggesting up to 20% misclassifications^39–41^. Guidelines have been issued^39,42,43^ but improving assessment precision remains a key issue^44^.

A most natural way to increase reliability is to enhance IHC-estimates by additional information, e.g. from gene expression. Methodological advances were reported for data normalization^45–48^ as well as for gene expression analysis^49–51^. A survey was given by Bolstad, Irizarry, Astrand and Speed^52^. Chen modelled the probability distributions for gene-expressions of ER, PGR and HER2 and set cutoff values at a posterior probability of 0.5 to discriminate receptor positive from negative patients^53^. Likewise, Laas set cutoffs for receptor positivity within the frequency distributions of gene-expression of ER and HER2^41^. Gong used random sampling to estimate optimum thresholds for ER and HER2 and verified these in a test cohort^54^. Bergqvist set thresholds for HER2 expression according to visual inspection^55^. Lopez introduced fuzzy rules to identify breast cancer biomarkers^56^. Concluding, prediction algorithms for low dimensional input space (such as hormone receptors), yield fairly similar results.

In previous papers^57,58^ we have worked on such approaches, applying standard statistical means (odds-products). In this work we expand the approach by drawing on Dempster Shafer decision Theory (DST)^59^.

DST has been widely applied in self driving cars^60–62^, driver’s vigilance monitoring^61^, aircraft technology^63,64^ and also in medical settings, e.g. image based decisions^65^, diagnosis of prostate^66^ and breast cancer^67^. In all these applications, unclear or even contradicting information from several sources are fused to arrive at decisions of optimized precision.

### Using decision theory for receptor status assessment

Classical probability theory characterizes the chance for an event to occur by a single number, its probability. A probability, *p,* characterizes the event as such, including its circumstances, but disregards the measuring process itself. The performance of the measuring process is characterized by sensitivity and specificity, in short by its receiver operating characteristics (ROC). In contrast, DST includes the measuring process and characterizes expected outcomes (say ‘receptor positive’, ‘Rez^+^’) by two numbers, the belief bel(Rez^+^) and the plausibility, pl(Rez^+^), together called ‘evidence’ of the outcome. Note that this definition of evidence specifically relates to the framework of decision theory and differs from more general meanings of the term, e.g. in ‘evidence based medicine’. The belief indicates the chance to correctly measure ‘positive’ by virtue and quality of the measuring process. The plausibility indicates the chance that the reading ‘positive’ (for an actually positive receptor) could also result (a) by chance or (b) represent a false positive outcome (for a receptor status truly ‘negative’). For each measurement with dichotomic outcome $\Omega = \left\{ {{\text{Rez}}^{ + } ,\;{\text{Rez}}^{ - } } \right\}$, ${\text{pl}}\left( {{\text{Rez}}^{ + } } \right) = 1 - {\text{bel}}\left( {{\text{Rez}}^{ - } } \right)$. DST additionally accommodates uncertainty, *θ*, defined as $\theta \left( {{\text{Rez}}^{ + } } \right){ = }\;{\text{pl}}\left( {{\text{Rez}}^{ + } } \right) - {\text{bel}}\left( {{\text{Rez}}^{ + } } \right) = 1 - {\text{bel}}\left( {{\text{Rez}}^{ - } } \right) - {\text{bel}}\left( {{\text{Rez}}^{ + } } \right)$, i.e. the uncertainty of an event is the difference between its plausibility and its belief. For brief notation we use ${\text{bel}}\left( {{\text{Rez}}^{ + } } \right) = \alpha$ and ${\text{bel}}\left( {{\text{Rez}}^{ - } } \right) = \beta$.

The main parts of this paper address readers familiar with DST and demonstrate how to combine several evidences for single hormone receptors and further combine several receptor estimates towards an overall hormone receptor status. Readers not yet familiar with DST may first see the ‘supplementary methods’ and then resume to read the following chapters.

## Materials and methods

### Data curated and used

In order to establish a comprehensive database^68^, we decided to re-use published gene expression data^69^ and screened the Gene Expression Omnibus (GEO)^70,71^ for breast cancer studies using the Affymetrix chip U133A + 2.0^72^. We found and curated 38 studies with 3753 samples.

Out of numerous methods available for normalization^46,73–76^ as compared by Bolstad^52^ and evaluated in our previous work^77^, we used RMA (MATLAB affyrma)^78^ and standardized data for each sample. Further batch corrections have been scrutinized in our previous work^77^ and were found ambiguous. We therefore refrained from performing them.

We adopted receptor genes and selected co-genes (see Table 1) from our previous paper^57^. Whereas in multiple logistic regression all genes would be incorporated simultaneously in the predictor (multiple logistic regression), the ODDS method performs logistic regressions on single genes and then combines the odds. In other words, in this former work, probabilities from IHC, gene and co-gene were joined according to conventional statistics, i.e. by multiplying odds. Hence we refer to this method as ‘ODDS’ in the following. Like most binary classifiers^79^, ODDS builds a score out of input variables (i.e. gene expression). This score is either compared to a threshold and the corresponding class assigned (crisp classification, + or −). Or else, the sharp threshold is replaced by an interval, within which the score is considered ‘uncertain’. We chose the latter possibility in the setup of ODDS and excluded samples classified ‘uncertain’. This lets the remaining samples (+ , −) gain certainty in their assessment, which is appropriate for a data basis on which our new discrimination method (DST) is evaluated.

**Table 1:** Receptor genes, co-genes and parameters from logistic regression.

			Logistic regression parameters		Logistic regression quality		Upper limits for beliefs	
		Probe set	$c_{0}$	$c_{1}$	Deviance of fit	N of samples	$\hat{\alpha }$	$\hat{\beta }$
**Estrogen**								
Gene	ESR1	205225_at	3.798	− 2.302	1106.4	2227	0.820	0.885
Co-gene	AGR3	228241_at	1.528	− 1.557	1254.1		0.790	0.841
**Progesterone**								
Gene	PGR	208305_at	0.886	− 1.944	1329.3	1714	0.755	0.660
Co-gene	ESR1	205225_at	4.112	− 2.422	845.0		0.826	0.878

Probe sets refer to the Affymetrix chip U133A + 2.0. For ‘deviance of fit’, see p.118 in McCullagh^81^. Note the double role of ESR1: It is the very receptor gene for ER but also serves as best co-gene for PGR, see our previous work^57^ regarding the selection of co-genes. Upper limits for beliefs $(\hat{\alpha },\hat{\beta })$ are explained in the next section (Eqs. 4–6).

When performing ODDS like in our previous paper, results may contradict IHC—for a few samples. As opposed to this, in the current work we took IHC-estimates for granted and applied ODDS only to samples where no IHC estimate was available. Hence, values imputed by ODDS could never contradict IHC-estimates—just to be on the save side. Moreover, ‘uncertain’ results from ODDS were also discarded. All in all, to establish the database for the present work, we retained results either from IHC or safely imputed by ODDS and call this procedure ‘sODDS’ (*safe*Odds).

**Figure 1:**
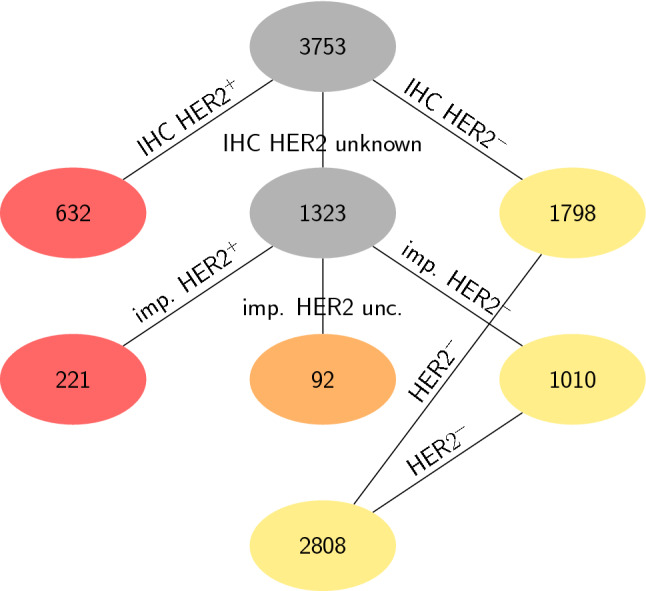
Selection of HER2-negative (HER2^−^) samples. The sODDS-method^57^ was applied to 3753 patient samples to extract those HER2^–^. If IHC receptor status was available, it was taken as is and samples accepted (1798 HER2^–^, yellow) or excluded (632 HER2^+^, red). Patients with unknown IHC-status (1323, grey) were accepted if HER2^–^ could be safely imputed (imp. HER2^-^) by the ODDS method (1010, yellow) and excluded if ODDS safely yielded HER2^+^ (221, red) or ‘uncertain’ (imp. HER2 unc., 92, beige). All in all, 1798 + 1010 = 2808 HER2^-^ patients resulted.

As first step of data cleansing we ruled out crosstalk by HER2: We dismissed HER2^+^ samples and accepted only those definitely assessed negative, either by IHC (1798 samples) or safely imputed via ODDS (1010 samples). For imputation we used default parameters and the threshold $\left| {score} \right| > \log \left( {\left( {1 - \varepsilon } \right)/\varepsilon } \right)$ with ${\varepsilon} = 0.15$, like in our previous work^57^. Finally, 1798 + 1010 = 2808 samples remained as HER2^-^, according to the sODDS method, see Fig. 1.

**Figure 2:**
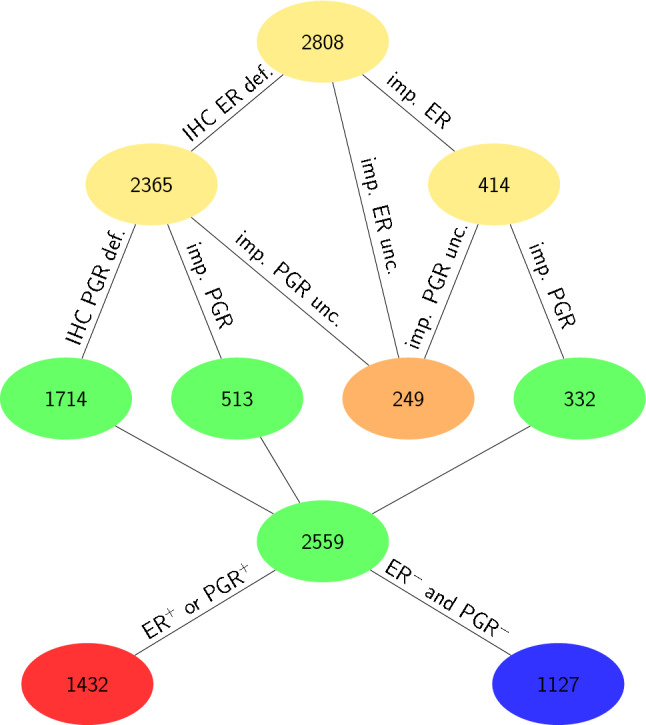
Selection of samples with definite hormone receptor status. Selection started from 2802 samples HER2^−^, see Fig. 1. If an IHC estimate for estrogen (ER) was available, ER was considered defined (ER def., 2365). Otherwise the ODDS method^57^ was applied, yielding either a safe result (imp. ER, 414) or resulting uncertain (imp. ER unc.), contributing to the set of 249 samples shown in beige. ODDS was applied to the set of 414 samples to impute progesterone status, either safely (imp. PGR, 332, green) or ending up uncertain (imp. PGR unc.), contributing to the beige set of 249. Finally, out of the 2365 samples with IHC available, IHC for progesterone was either available (IHC PGR def., 1714, green), safely imputed by ODDS (imp. PGR, 513, green) or uncertain (imp. PGR unc.), again contributing to the beige set. All in all, IHC receptor status was available for 1714 samples and safely imputed for 513 + 332 = 845 samples. This resulted in 2559 samples with definite hormone receptor status, out of which 1432 were positive (red) and 1127 negative (blue). These constituted the input set for DST, as shown in Fig. 7. Note that no samples exist with PGR_IHC_ defined and ER_IHC_ imputed.

These 2808 samples were then checked regarding ER and PGR as follows: If IHC-estimates were available for ER as well as PGR, samples were taken as is (1714 samples). If IHC was only available for ER and the status of PGR could be safely imputed via ODDS^57^ with sufficient score (see above), samples were also included (513 samples). If IHC was missing for both, ER as well as PGR, samples were taken only if both status could be imputed safely via ODDS (332 samples). Thus, 1714 + 513 + 332 = 2559 samples were retained with receptor status established most accurately by state of the art (IHC plus ODDS ≙ sODDS), see Fig. 2. We used only these to evaluate the benefit of applying decision theory.

### Survival rates and quality of previous receptor status assessments

**Figure 3:**
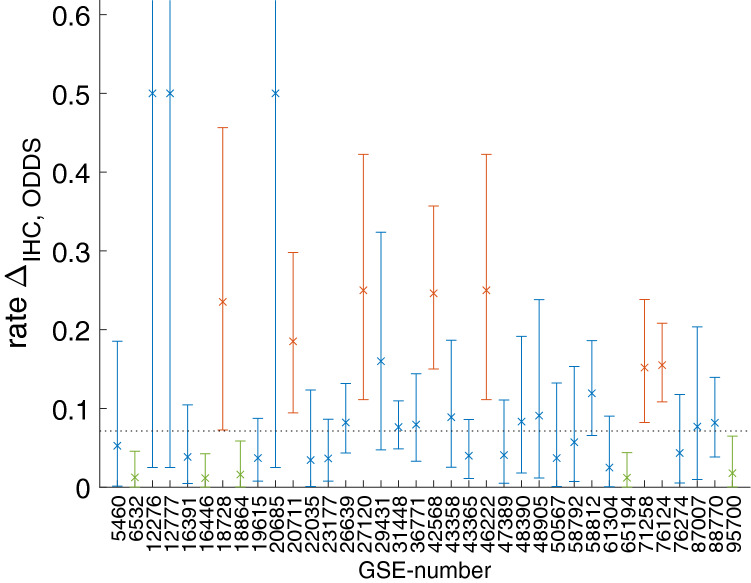
Control chart for estrogen receptor status: differences between IHC and ODDS gene expression estimates. x-axis: label of study. y-axis: rate of difference. Dotted line: overall mean rate of differences between IHC and ODDS *p* = 159/2227 = 0.071, see SuppTable 2. Markers (x) within error bars: rate of discordant samples in respective study. Lower and upper bounds of error bars denote 95% confidence limits. If the upper bound lies below the dotted line, the respective study has a discrepancy rate significantly below average (green). If the lower bound lies above the dotted line, the respective study has a discrepancy rate significantly above average (red).

A study-wise evaluation of receptor status assessment via IHC and ODDS was performed, see Supp. Table 2. The concordance of receptor status between IHC and ODDS is much better for ER than for PGR, due to larger variance of ER in gene expression data. Results were further elaborated in a control chart, see Fig. 3. The dotted line represents *p*, the overall average rate of differences, computed from Supp. Table 2 as *p* = 159 / 2227 ~ 0.071. Markers within bars represent expectation values (Δ_IHC, ODDS_ + 1)/(N_IHC_ + 2) of discordance rates derived from the beta-distribution, see the mathematical formalism in section ‘Concordance between IHC and gene expression’ in supplementary materials. Bars denote 95% confidence intervals of the respective mean values, allowing the qualification of studies against each other: If an upper bound lies below the dotted line in Fig. 3, the respective study has a discrepancy rate significantly below average (green). If a lower bound lies above the dotted line, the discrepancy rate of that study is significantly above average (red).

Similarly, a control chart for progesterone is shown in Supp. Fig. 2.

Additionally, we evaluated the scmgene-marker (from package genefu^80^) for estrogen only, since scmgene does not give PGR estimates. Note that scmgene was evaluated for all (3753) samples but results were only compared for those 2559 with HER2^−^ and reliable hormone receptor status, see Supp. Table 1.

**Figure 4:**
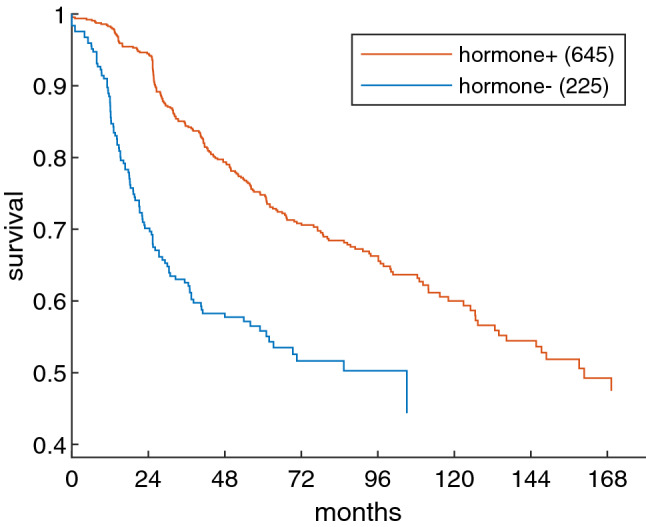
Survival free from recurrence for hormone status. Hormon status is considered positive, if either ER or PGR are positive, otherwise negative. Kaplan Meier estimates of survival free from recurrence for 870 patients (who had rfs-data) for different hormone receptor status. Log-rank test *p* = 4.2e–06.

Survival has been related to receptor status for ER (Supp. Fig. 3), PGR (Supp. Fig. 4) and hormone overall, i.e. either estrogen or progesterone being positive, see Fig. 4.

### Responsibility functions for gene expression

In order to compute DST evidences from gene expression, we will first define responsibility functions as a prerequisite. The concept was coined by Hastie^79^ for weighing two distributions against each other regarding membership of a measurement in question. We use this for distributions of gene-expression data and their indication of receptor status. Based on these, ‘mass functions’ will be computed, and these converted into evidence:

Gene expression + IHC → responsibility functions (→ mass functions) → evidence.

In the main part of this paper for brevity we skip the concept of masses (hence above shown in parenthesis) and present formulae for evidence directly based on responsibility functions. Masses are relegated to supplementary methods.

Responsibility functions are exemplified now for the expression of *one* gene, labelled ‘Expr', to keep notation slim. It applies analogously to the other genes considered.

**Figure 5:**
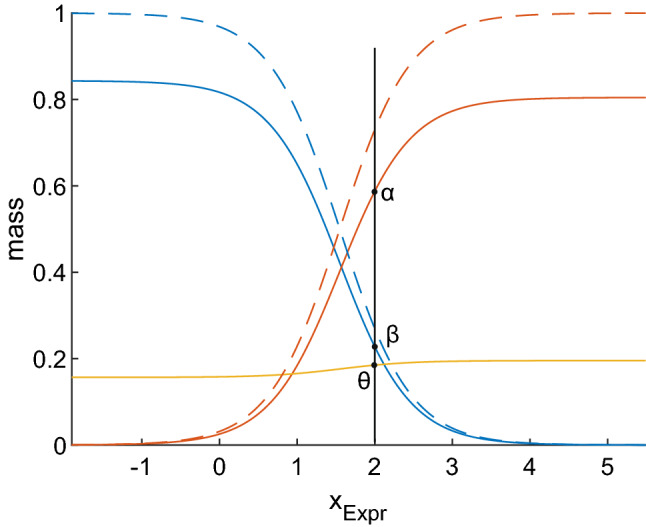
Decision theory evidences obtained from logistic regression. A logistic regression of IHC receptor status (IHC^+^ ≙ 1, IHC^-^ ≙ 0) versus gene expression $(x_{{{\text{Expr}}}} )$ was performed to obtain the responsibility function for receptor positivity r_+_ (dotted red curve) and r_-_ (dotted blue). It will be shown later (Eq. 6) that r_+_ has to be multiplied by an upper limit, $\widehat{\alpha }_{{{\text{IHC}}}}$, to obtain the actual belief $\alpha_{{{\text{Expr}}}} \left( {x_{{{\text{Expr}}}} } \right) = \widehat{\alpha }_{{{\text{IHC}}}} \cdot r_{ + } \left( {x_{{{\text{Expr}}}} } \right)$, see the solid red curve. Likewise $\beta_{{{\text{Expr}}}} \left( {x_{{{\text{Expr}}}} } \right) = \widehat{\beta }_{{{\text{IHC}}}} \cdot r_{ - } \left( {x_{{{\text{Expr}}}} } \right)$ (solid blue). Uncertainty: ochre. For a given expression value, e.g. $x_{{{\text{Expr}}}}$ = 2, one can read off belief in positive (*α*), belief in negative (*β*) and uncertainty (*θ*).

We construct the responsibility function, r_+_, for receptor positivity from logistic regression (fitting coefficients: c_0_, c_1_) of IHC receptor status versus gene expression, *x*_Expr_, see Eq. (1) and the increasing dotted red line in Fig. 5. The (dual) responsibility function, ${\text{r}}_{ - }$, for receptor-negativity is simply given by ${\text{r}}_{ - } = 1 - {\text{r}}_{ + }$, see Eq. (1) and the decreasing blue dotted line in Fig. 5.

1 $$\begin{aligned} {\text{r}}_{ + } \left( {x_{{{\text{Expr}}}} \left| {c_{0} ,c_{1} } \right.} \right) & = \frac{{\exp \left( {c_{0} + c_{1} x_{{{\text{Expr}}}} } \right)}}{{1 + \exp \left( {c_{0} + c_{1} x_{{{\text{Expr}}}} } \right)}} \\ {\text{r}}_{ - } \left( {x_{{_{{{\text{Expr}}}} }} \left| {c_{0} ,c_{1} } \right.} \right) & = 1 - {\text{r}}_{ + } \left( {x_{{{\text{Expr}}}} \left| {c_{0} ,c_{1} } \right.} \right) \\ \end{aligned}$$

Logistic regression is carried out separately for estrogen receptor gene (ESR1), estrogen co-gene (AGR3), progesterone receptor gene (PGR) and its co-gene (ESR1). Results are consolidated in Table 1.

### Obtaining evidences

**Figure 6:**
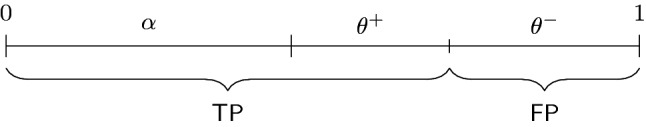
Estimating an upper bound for belief in receptor positivity. True positives (TP) and false positives (FP) can be used to derive a first equation in $\alpha_{{{\text{Expr}}}}$ and $\theta_{{{\text{Expr}}}}$ (Eq. 3). A second equation may be deduced by including TN and FN, Eq. (2). Both can be solved to estimate $\alpha_{{{\text{Expr}}}}$ and $\theta_{{{\text{Expr}}}}$. From these follow upper bounds for the belief in positivity ($\hat{\alpha }_{{{\text{Expr}}}}$) and negativity ($\hat{\beta }_{{{\text{Expr}}}}$), Eqs. (4 and 5), respectively.

We define DST masses (see supplementary methods) and evidences by drawing on responsibility functions as follows: We observe in Fig. 5 that ${\text{r}}_{ + } \to 1$ as gene expression approaches its maximum. However, not even maximum gene expression in reality provides total certainty of receptor status being positive. To account for this fact, the responsibility function has to be multiplied by an upper bound, $\hat{\alpha }_{{{\text{Expr}}}}$, for the belief in ‘ + ’. Similarly $\hat{\beta }_{{{\text{Expr}}}}$ acts as upper bound for the belief in ‘−’. Next we show how $\hat{\alpha }_{{{\text{Expr}}}}$ and $\hat{\beta }_{{{\text{Expr}}}}$ are obtained, see Fig. 6.

Out of all positive IHC-measurements (represented by the interval (0,1)), a certain fraction has been confirmed positive by gene expression, due to virtue of the method. This fraction of measurements is represented by the interval (0,$\alpha_{{{\text{Expr}}}}$) as part of the interval (0,1). Given a positive IHC measurement, the plausibility $\beta_{{{\text{Expr}}}} = 0$, and hence the rest of the interval (0,1) represents nothing but uncertainty according to DST: $\theta_{{{\text{Expr}}}} = 1 - \alpha_{{{\text{Expr}}}} - 0$. As depicted in Fig. 6, some fraction, *θ*^+^, of uncertain predictions from gene expression will result positive merely by chance, not by virtue of the method. Both parts together represent all true positive IHC-measurements: $\alpha_{{{\text{Expr}}}} + \theta_{{{\text{Expr}}}}^{ + } = TP$. A second part of uncertainty, $\theta_{{{\text{Expr}}}}^{ - }$, will represent erroneously assessed false positives (*FP*), although being truly negative samples. Based on this nomenclature we proceed as follows:

Consider all samples qualified ‘uncertain’ by gene expression, see Fig. 6. The part $\theta_{{{\text{Expr}}}}^{ + }$ represents truly positive samples, whereas $\theta_{{{\text{Expr}}}}^{ - }$ are truly negative ones. We may now assume on good grounds that the ratio ${{\theta_{{{\text{Expr}}}}^{ + } } \mathord{\left/ {\vphantom {{\theta_{{{\text{Expr}}}}^{ + } } {\theta_{{{\text{Expr}}}}^{ - } }}} \right. \kern-\nulldelimiterspace} {\theta_{{{\text{Expr}}}}^{ - } }}$ among the uncertain ones from gene expression is similar (equal) to the ratio of positives and negatives found by IHC, see also Shoyaib^82^. Considering the contingency table of IHC-measurements, note that positive measurements comprise $TP + FN$, whereas negative ones are composed as $TN + FP$. Hence we may put

2 $$\frac{{\theta_{{{\text{Expr}}}}^{ + } }}{{\theta_{{{\text{Expr}}}}^{ - } }} = \frac{TP + FN}{{TN + FP}}$$

Moreover, the fraction of gene expression measurements being positive by virtue or chance, $\alpha_{{{\text{Expr}}}} + \theta_{{{\text{Expr}}}}^{ + }$, is set equal to the fraction of IHC-true positives:

3 $$\alpha_{{{\text{Expr}}}} + \theta_{{{\text{Expr}}}}^{ + } = \frac{TP}{{TP + FP}}$$

Since $\alpha_{{{\text{Expr}}}} + \theta_{{{\text{Expr}}}}^{ + } + \theta_{{{\text{Expr}}}}^{ - } = 1$, Eqs. (2 and 3) can now be solved to yield the maximum possible belief in positive receptor status (derived from the gene in question):

4 $$\hat{\alpha }_{{{\text{Expr}}}} = \frac{TP \cdot TN - FP \cdot FN}{{\left( {TP + FP} \right) \cdot \left( {TN + FP} \right)}}$$

Likewise we obtain for the maximum belief in negative status:

5 $$\hat{\beta }_{{{\text{Expr}}}} = \frac{TP \cdot TN - FP \cdot FN}{{\left( {TN + FN} \right) \cdot \left( {TP + FN} \right)}}$$

Considering these maximum beliefs, we finally obtain for the evidence derived from gene expression, for illustration see Fig. 5:

6 $$\begin{aligned} \alpha_{{{\text{Expr}}}} \left( {x_{{{\text{Expr}}}} } \right) & = \hat{\alpha }_{{{\text{Expr}}}} \cdot r_{ + } \left( {x_{{{\text{Expr}}}} \left| {c_{0} ,c_{1} } \right.} \right) \\ \beta_{{{\text{Expr}}}} \left( {x_{{{\text{Expr}}}} } \right) & = \hat{\beta }_{{{\text{Expr}}}} \cdot r_{ - } \left( {x_{{{\text{Expr}}}} \left| {c_{0} ,c_{1} } \right.} \right) \\ \end{aligned}$$

Note that the whole formalism (Eqs. 1–6) is performed separately for each gene (and co-gene), using the data of corresponding IHC measurements. For generality and brevity of above notation, the subscript ‘Expr’ stands for each of those genes.

### Completing the decision theory framework

With responsibility functions and evidences available we may now assemble the decision framework as follows.

Each hormone receptor (ER, PGR) is assessed via three sources of information, each yielding separate evidence (in our case of dichotomous data: 2 numbers):

1. IHC → evidence $$(\alpha_{{{\text{IHC}}}} ,\;\beta_{{{\text{IHC}}}} )$$
2. gene expression of the receptor gene → evidence $(\alpha_{{{\text{Gen}}}} ,\;\beta_{{{\text{Gen}}}} )$
3. gene expression of a co-gene → evidence ($\alpha_{{{\text{Co}}}}$,$\beta_{{{\text{Co}}}}$)

In a first step, evidences for the receptor gene ($\alpha_{{{\text{Gen}}}}$,$\beta_{{{\text{Gen}}}}$) and its co-gene ($\alpha_{{{\text{Co}}}}$,$\beta_{{{\text{Co}}}}$) are combined by the evidence combination rule (ECR) introduced by Dempster^83^ (labelled ‘$\oplus_{{\text{D}}}$’) to obtain joint evidence ($\alpha_{{{\text{Expr}}}}$,$\beta_{{{\text{Expr}}}}$) from gene expression:

7 $$\left( {\alpha_{{{\text{Expr}}}} ,\beta_{{{\text{Expr}}}} } \right) = \left( {\alpha_{{{\text{Gen}}}} ,\beta_{{{\text{Gen}}}} } \right) \oplus_{{\text{D}}} \left( {\alpha_{{{\text{Co}}}} ,\beta_{{{\text{Co}}}} } \right)$$

As explained in supplementary methods, the operation $\oplus_{{\text{D}}}$ yields in detail:

8 $$\begin{gathered} \alpha_{{{\text{Expr}}}} = \frac{{\alpha_{{{\text{Gen}}}} \alpha_{{{\text{Co}}}} + \theta_{{{\text{Gen}}}} \alpha_{{{\text{Co}}}} + \alpha_{{{\text{Gen}}}} \theta_{{{\text{Co}}}} }}{{1 - \alpha_{{{\text{Gen}}}} \beta_{{{\text{Co}}}} - \beta_{{{\text{Gen}}}} \alpha_{{{\text{Co}}}} }} \\ \beta_{{{\text{Expr}}}} = \frac{{\beta_{{{\text{Gen}}}} \beta_{{{\text{Co}}}} + \theta_{{{\text{Gen}}}} \beta_{{{\text{Co}}}} + \beta_{{{\text{Gen}}}} \theta_{{{\text{Co}}}} }}{{1 - \alpha_{{{\text{Gen}}}} \beta_{{{\text{Co}}}} - \beta_{{{\text{Gen}}}} \alpha_{{{\text{Co}}}} }} \\ \theta_{{{\text{Expr}}}} = 1 - \alpha_{{{\text{Expr}}}} - \beta_{{{\text{Expr}}}} = \\ = \frac{{\theta_{{{\text{Gen}}}} \theta_{{{\text{Co}}}} }}{{1 - \alpha_{{{\text{Gen}}}} \beta_{{{\text{Co}}}} - \beta_{{{\text{Gen}}}} \alpha_{{{\text{Co}}}} }} \\ \end{gathered}$$

This evidence for gene expression is—in a second step—combined with the evidence from IHC, ($\alpha_{{{\text{IHC}}}}$,$\beta_{{{\text{IHC}}}}$), either using rule ‘$\oplus_{{\text{D}}}$’ once more or, alternatively the ECR defined by Yager^83^, labelled ‘$\oplus_{{\text{Y}}}$’. Since $\oplus_{{\text{Y}}}$ more easily accommodates contradicting evidence (from IHC and gene expression), we opt for $\oplus_{{\text{Y}}}$ and obtain:

9 $$\begin{aligned} \alpha_{{{\text{Rez}}}} & = \alpha_{{{\text{Expr}}}} \alpha_{{{\text{IHC}}}} + \theta_{{{\text{Expr}}}} \alpha_{{{\text{IHC}}}} + \alpha_{{{\text{Expr}}}} \theta_{{{\text{IHC}}}} \\ \beta_{{{\text{Rez}}}} & = \beta_{{{\text{Expr}}}} \beta_{{{\text{IHC}}}} + \theta_{{{\text{Expr}}}} \beta_{{{\text{IHC}}}} + \beta_{{{\text{Expr}}}} \theta_{{{\text{IHC}}}} \\ \theta_{{{\text{Rez}}}} & = \theta_{{{\text{Expr}}}} \theta_{{{\text{IHC}}}} + \alpha_{{{\text{Expr}}}} \beta_{{{\text{IHC}}}} + \beta_{{{\text{Expr}}}} \alpha_{{{\text{IHC}}}} \\ \end{aligned}$$

In supplementary methods we explain in detail how general concepts of DST (capable of handling a set of multiple outcomes) boil down to Eqs. (8 and 9) in case of just two outcomes, receptor status $\left\{ {^{\prime} + ^{\prime},\;^{\prime} - ^{\prime}} \right\}$.

The above procedure is carried out similarly to obtain the combined evidence for the PGR-status ($\alpha_{{{\text{PGR}}}}$,$\beta_{{{\text{PGR}}}}$). Note that notation is turned from general to specific in the following ($\alpha_{{{\text{Rez}}}} \to \alpha_{{{\text{ER}}}}$ and $\alpha_{{{\text{Rez}}}} \to \alpha_{{{\text{PGR}}}}$, respectively).

In a last step, the clinical decision for ‘hormone versus chemo’ is modelled along the lines of DST. Clinically, a patient is considered receptor positive (and will receive hormone therapy) if either ER or PGR (or both) are positive. Classically, this is a crisp, logical decision as stated. DST however, allows more elaborate combination rules to combine evidences for estrogen ($\alpha_{{{\text{ER}}}}$,$\beta_{{{\text{ER}}}}$) and progesterone ($\alpha_{{{\text{PGR}}}}$,$\beta_{{{\text{PGR}}}}$).

Thus, considering ER as well as PGR, each being assessed by IHC, gene and co-gene, performing some algebra (detailed in supplementary methods) yields the overall evidence for ‘hormone receptor status’ $\left( {\alpha_{{\text{H}}} = {\text{bel}}_{{\text{H}}} (pos),\;\beta_{{\text{H}}} = {\text{bel}}_{{\text{H}}} (neg)} \right)$:

10 $$\begin{aligned} \alpha_{{\text{H}}} & = \alpha_{{{\text{ESR}}}} + \alpha_{{{\text{PGR}}}} - \alpha_{{{\text{ESR}}}} \alpha_{{{\text{PGR}}}} \\ \beta_{{\text{H}}} & = \beta_{{{\text{ESR}}}} \cdot \beta_{{{\text{PGR}}}} \\ \end{aligned}$$

As always we have to accept an amount of uncertainty given by $\theta_{{\text{H}}} = 1 - \alpha_{{\text{H}}} - \beta_{{\text{H}}}$, indicating hormone receptor status being indeterminable. From these evidences, a most reasonable decision rule can be derived:

11 $$\begin{aligned} & {\text{Hormone receptor positive}} \leftrightarrow \alpha_{{\text{H}}} > \beta_{{\text{H}}} + \theta_{{\text{H}}} \\ & {\text{Hormone receptor negative}} \leftrightarrow \beta_{{\text{H}}} > \alpha_{{\text{H}}} + \theta_{{\text{H}}} \\ & {\text{Hormone receptor indeterminable}} \leftrightarrow \left| {\alpha_{{\text{H}}} - \beta_{{\text{H}}} } \right| \le \theta_{{\text{H}}} \\ \end{aligned}$$

In our simple case with only two outcomes these criteria reduce to $\alpha_{{\text{H}}} > 0.5$ for definitely receptor positive and $\beta_{{\text{H}}} > 0.5$ for definitely receptor negative.

## Results

### DST results for patient cohort

In our previous work^57^, we considered receptors (ER, PGR) separately. For each of them routine estimates (based on IHC only, 1714 samples, see Fig. 2) could be significantly improved by adding information from gene expression via conventional statistics (odds ratio) and we label this former method ‘ODDS’. Note that for patients lacking one or both IHC estimates, receptor status was imputed from gene expression according to ODDS, including an uncertainty region, as in our previous paper. However, to obtain a dataset of optimum quality for the present work, only samples rendering safe imputations were retained (513 + 332 = 845), see Fig. 2.

**Figure 7:**
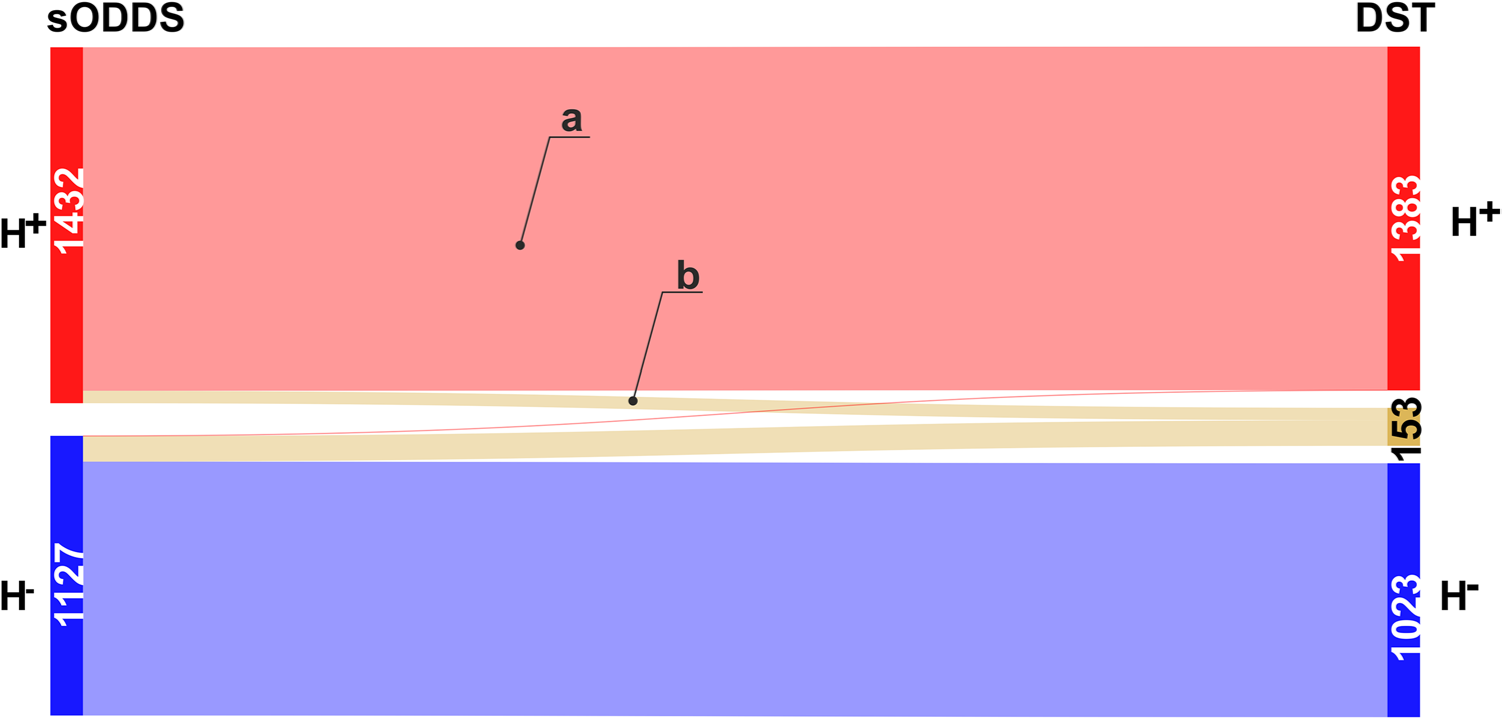
Alluvial flow diagram for patients originally diagnosed by ODDS and then checked by DST. Left margin: Hormone receptor assessed by method sODDS: positive (H^+^) and negative (H^−^). A sample was considered H^+^ if at least one receptor was positive. Lacking IHC estimates were imputed by ODDS and included only if imputation was safe, otherwise discarded. Hence, no patients with receptor status ‘undetermined’ appear in dataset subjected to DST. Right margin: Evaluation by DST confirms the vast majority of estimates from sODDS but leaves some 153 as undetermined (ochre colored). Only one patient is definitely considered false negative and changed to positive.

Then we demonstrated that further improvement in receptor assessment can be achieved by applying decision theory. Besides yielding new estimates for each receptor, DST allows to combine estimates for ER and PGR into a joined ‘hormone’ receptor estimate. Based on Eqs. (10 and 11), hormone receptor status was newly predicted by DST for each patient, see the contingency Table 2 and Fig. 7.

**Table 2:** Receptor status due to decision theory (DST) compared with previous method (ODDS).

		Method DST			
		−	Uncertain	+	∑
Method sODDS	−	1023	103	1	1127
	+	0	50	1382	1432
∑		1023	153	1383	2559

2559 Patients have been assessed for hormone receptor status by method sODDS to comprise the input dataset. DST was then applied to this dataset. A sample was considered H^+^ if at least one receptor was positive. Difference between methods sODDS and DST is reflected in Cohen’s *κ* = 0.88. Note that uncertain results were considered as separate category in computing *κ.*

To demonstrate the clinical benefit of DST, we consider two groups:

1. Patients diagnosed receptor positive by sODDS, and being confirmed by DST. They have correctly received hormone treatement, see the flow labelled ‘a’ in Fig. 7.
2. Patients diagnosed receptor positive by sODDS, but being questioned by DST, see flow ‘b’. They have very probably also received hormone treatment but it remained ineffective. At the same time they might have been deprived of life-saving chemo.

**Figure 8:**
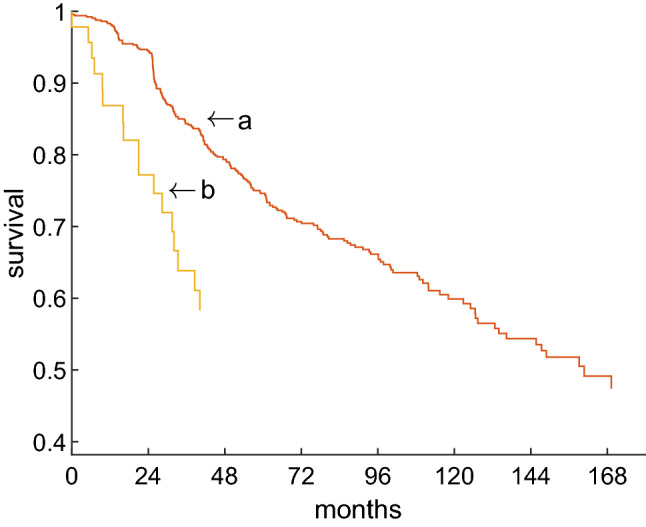
Precise versus inadequate treatment based on hormone receptor status. Survival free from recurrence, according to Kaplan–Meier. Red: Patients treated correctly as receptor positive (flow (a) in Fig. 7). Ochre: Patients erroneously assigned positive (flow (b) in Fig. 7).

Figure 8 shows a strikingly worse survival of patients assumed receptor positive although being negative (logrank–Wilcoxon *p* = 0.009). Note that out of 1432 patients considered positive by sODDS, only for 651 survival data were available. Among these, 26 were questioned by DST. Nonetheless the difference was statistically significant at the 1%-level.

### DST compared to ODDS

It is interesting to see what DST achieves as compared to ODDS when starting from the same patient cohort. We therefore took the same sample of patients (2559) as in Table 2 and Fig. 7, with 1432 receptor status positive and 1127 negative according to sODDS. Instead of DST we this time applied the ODDS method, including a uncertainty region, see Table 3.

**Table 3:** Receptor status according to ODDS.

		Method ODDS			
		−	Uncertain	+	∑
Method sODDS	−	1035	52	40	1127
	+	31	30	1371	1432
∑		1066	82	1411	2559

2559 Patients have been assessed for hormone receptor status by sODDS to comprise the input dataset. A sample was considered H^+^ if at least one receptor was positive. Difference in methods is reflected in Cohen’s *κ* = 0.88.

Most strikingly, DST had flagged almost double as many patients ‘uncertain’ (156, see Table 2) as compared to ODDS (82), although the very same safety threshold was used. This clearly reflects an advantage of DST, since patients with questionable status would be re-evaluated before assigning the adequate therapy.

To characterize these differences in judgement we performed a face-to-face comparison between DST and ODDS on the very same input dataset, see Table 4. Samples additionally labelled ‘uncertain’ by DST originate to almost equal parts from negative (44) and positive (39) estimates according to ODDS. Only very few samples considered uncertain by ODDS, migrate to negative (1) or positive (11) according to DST.

**Table 4:** DST Decision theory compared to ODDS.

		Method DST			
		−	uncertain	+	∑
Method ODDS	−	1022	44	0	1066
	Uncertain	1	70	11	**82**
	+	0	39	1372	1411
∑		1023	**153**	1383	2559

The same dataset (2559 samples) was subjected to DST and ODDS, both including the same uncertainty regions as described. DST is seen to flag 153 patients as uncertain, i.e. of questionable hormone receptor status. As compared to ODDS almost double as many patients are detected to be re-checked before therapies are assigned. Difference in methods is reflected in Cohen’s *κ* = 0.88.

## Discussion

Dempster Shafer decision theory is a very potent framework. Its strength lies in combining information from several sources about the same item (here exemplified by IHC, Gene and Co-Gene estimates for one receptor) and also evidences for several different items, interacting in a biological setting (here exemplified by estrogen and progesterone regarding precision therapy of breast cancer).

We have started from some best methodology available up to now (sODDS), i.e. receptor status assessment via IHC enhanced by gene expression. ODDS had already been shown to improve precision therapy^57^, and here we demonstrate that further improvement is possible by application of DST. Since absolute truth of receptor status is unknown, the usefulness of our approach is demonstrated by disease free survival being severely degraded in patients flagged by DST as possibly wrongly diagnosed and treated. Explicit data on treatment are rare in the breast cancer studies downloaded from GEO. Hence we performed our calculations on the reasonable assumption that treatment was given according to the best knowledge available about receptor status.

The potency of DST is further underpinned by directly comparing both methods applied to the same dataset: DST flags about twice as many patients as ‘uncertain’ (153) as ODDS (82). Additional patients subjected to a re-evaluation of hormone receptor status will improve precision of therapy.

Formulae and results derived therefrom incorporate three assumptions which we think were reasonable:

To compute basic belief assignments for each patient, we obtained DST mass functions by logistic regression. It proved more stable than other possible methods, e.g. fitting bimodal distributions such as Gaussian mixtures, as done in a previous study^58^.

For joining evidences from 2 genes for the same receptor we chose the Dempster ECR. For adding IHC evidence to those from gene expression, we opted for the Yager ECR.

To obtain upper limits $\hat{\alpha }$,$\hat{\beta }$ for belief we reasonably assumed that the fraction of positive results by virtue or chance equals the fraction of IHC-true positives (Eq. 3). Likewise we assumed that the ratio of uncertain positive and negative cases equals the ratio of positives and negatives (Eq. 2).

It is interesting to note that up to now clinical decisions follow the conventional (Cantor) non-exclusive OR-logics: ‘If ER or PGR receptor estimates is/are positive, the patient is considered positive’. In part, the strength of DST originates from elaborate evidence joining: Simple and/or combinations (such as estrogen-or-progesterone receptor positivity) are fundamentally refined, since DST draws on *two* numbers (belief and plausibility) in each decision, instead of just a single number as conventional probability theory does. In addition, information from different sources is joined according to mathematical rules rather than ‘clinical intuition’.

**Figure 9:**
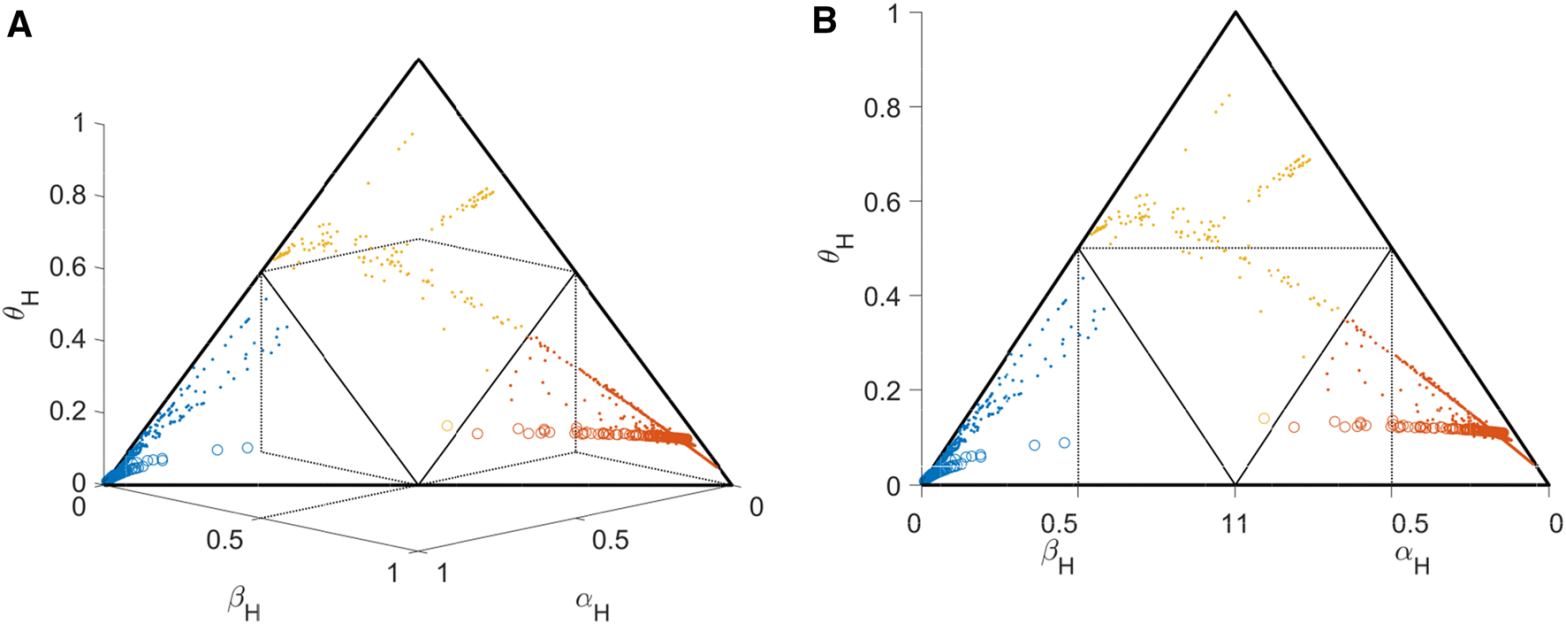
Decision space of hormone receptor status according to DST. (**A**) The three evidences of receptor status, belief in positive (*α*_H_), belief in negative (*β*_H_) and uncertainty (*θ*_H_), of all samples shown in a three dimensional plot. Colors indicate receptor assignment according to DST (positive ≙ red, negative ≙ blue, uncertain ≙ ochre). Dots: assignment via available IHC. Open circles: due to lacking IHC, assignment by imputation via DST. Decision borders according to Eq. (11) are indicated by dotted lines at *α*_H_ = 0.5, *β*_H_ = 0.5 and *θ*_H_ = 0.5. Samples with same status assigned appear confined within the same respective tetrahedral space. Note that all points lie in one plane due to the definition (*α*_H_ + *β*_H_ + *θ*_H_ = 1, see SuppEq. 16) within a triangle (surrounded by heavy lines). (**B**) The triangle plane in panel (A) seen from a slightly different aspect, along a vector parallel to plane (*α*_H_ *.β*_H_), and perpendicular to the baseline of the triangle. Decision borders are more clearly visible in this aspect. In this projection the point (*α*_H_ = 1*, β*_H_ = 1) in the foreground of panel (A) coincides with the baseline of the triangle, and so do the axes *α*_H_ and *β*_H_. Note that they run in opposite directions. Hormone receptor positive samples lie exclusively in the lower right triangle, negative ones in the lower left whereas uncertain results in the kite-shaped area.

Joining evidences from both receptors in a more intricate way according to DST, allows for an intuitively convincing view on the classification, see Fig. 9. From the definition of *θ*_H_ = 1−*α*_H_−*β*_H_ follows *α*_H_ + *β*_H_ + *θ*_H_ = 1 and, accordingly, DST beliefs (*α*_H_, *β*_H_, *θ*_H_ ) of all samples lie in one single plane in 3-dimensions, cutting through the axes at *α*_H_ = 1, *β*_H_ = 1 and *θ*_H_ = 1, see panel A. Decision boundaries (dotted lines in Fig. 9) originate at 0.5 on each axis of evidence (*α*_H_ = 0.5, *β*_H_ = 0.5) and confine tetrahedrons, each containing exclusively positive (red) or negative (blue) samples, respectively. These tetrahedrons appear as equilateral triangles in the view shown in panel B. Uncertain samples (shown in ochre) appear within the pentahedron (panel A), which reduces to a kite-shaped area in the view shown in panel B.

While in this paper we have presented a very simple example to demonstrate feasibility and benefit of the method, possible applications are wide in scope. Pathology and laboratory medicine in many cases have to deliver crisp decisions even if measurements by complementary methods lack agreement. DST is the method of choice to handle such situations.

The final evidence obtained by DST also allows assigning weights (loss functions) to certain outcomes in order to model clinical consequences (Eq. 11). Suppose, for example, a false positive outcome entails much more detrimental consequences than a false negative does.

Several different methods are available for joining: The ‘Dempster additivity rule’, $\oplus_{{\text{D}}}$, works best with rather concordant variables. Needless to mention that correlation must not be too close to unity, as in this case the second variable would not add any significant information to the first one. As opposed, the ‘Yager rule’, $\oplus_{{\text{Y}}}$, lends itself to join evidences from rather contradicting sources.

OMICS data often comprise a considerable number of estimates, say counts for multiple fragments out of a gene, needing to be condensed to characterize the gene (activity) as a whole. A plethora of similar situations can rigorously be handled by DST. Even if some of these estimates should be (partly) contradictive, DST is an appropriate tool for consolidating. Formulae need to be adapted to the particular situation in question.

We think that this work may help pave the way for decision theory entering OMICS data science.

## Supplementary Information

Supplementary Information.
