# Supplementary material for: Decision theory for precision therapy of breast cancer
Source: Sci Rep. 2021 Feb 19;11:4233. doi: 10.1038/s41598-021-82418-7 (PMC7895957; doi:10.1038/s41598-021-82418-7)
Supplement: Supplementary file 1 — Supplementary Information. [file 41598_2021_82418_MOESM1_ESM.pdf]

Supplementary Material for Decision theory for precision therapy of breast cancer

Michael Kenn[1], *Dan Cacsire Castillo-Tong*[2], *Christian F. Singer*[2], *Rudolf Karch*[1], *Michael Cibena*[1], *Heinz Koelbl*[3], *and Wolfgang Schreiner*[1*]

[1] Section of Biosimulation and Bioinformatics
Center for Medical Statistics, Informatics and Intelligent Systems (CeMSIIS)
Medical University of Vienna, Spitalgasse 23, A-1090 Vienna, Austria

[2] Translational Gynecology Group, Department of Obstetrics and Gynecology, Comprehensive Cancer Center, Medical University of Vienna, Waehringer Guertel 18-20, A-1090 Vienna, Austria

[3] Department of General Gynecology and Gynecologic Oncology, Medical University of Vienna, Waehringer Guertel 18-20, A-1090 Vienna, Austria

[*] Correspondence should be addressed to Wolfgang Schreiner
wolfgang.schreiner@meduniwien.ac.at

In this section we give a general and more formal outline of **D**empster **S**hafer decision **T**heory (DST), [1-4], starting with a tutorial example (SuppFig. 1) that may be skipped by readers familiar with DST. Formulae given in the results section of this paper have been deduced from this framework for the specific structure of data considered here (hormone receptors). Along the same lines, formulae and evidences can be deduced for any other structure of data.

Tutorial example for one source of information with multiple outcomes

Let $\Omega = \{A_1, A_2, ..., A_n\}$ be a set of n different, mutually exclusive *events*, also called 'frame' (of discernment) of one source of information. DST does not assign a probability to each event (as conventional statistics does) but also considers the fact that the outcome of a measuring process may not be exactly one of those possible events.

First we consider measurements of full precision, yielding exactly one specific event as their outcome, see the bottom part of SuppFig. 1 ('full precision measurements', in DST also called 'singletons').

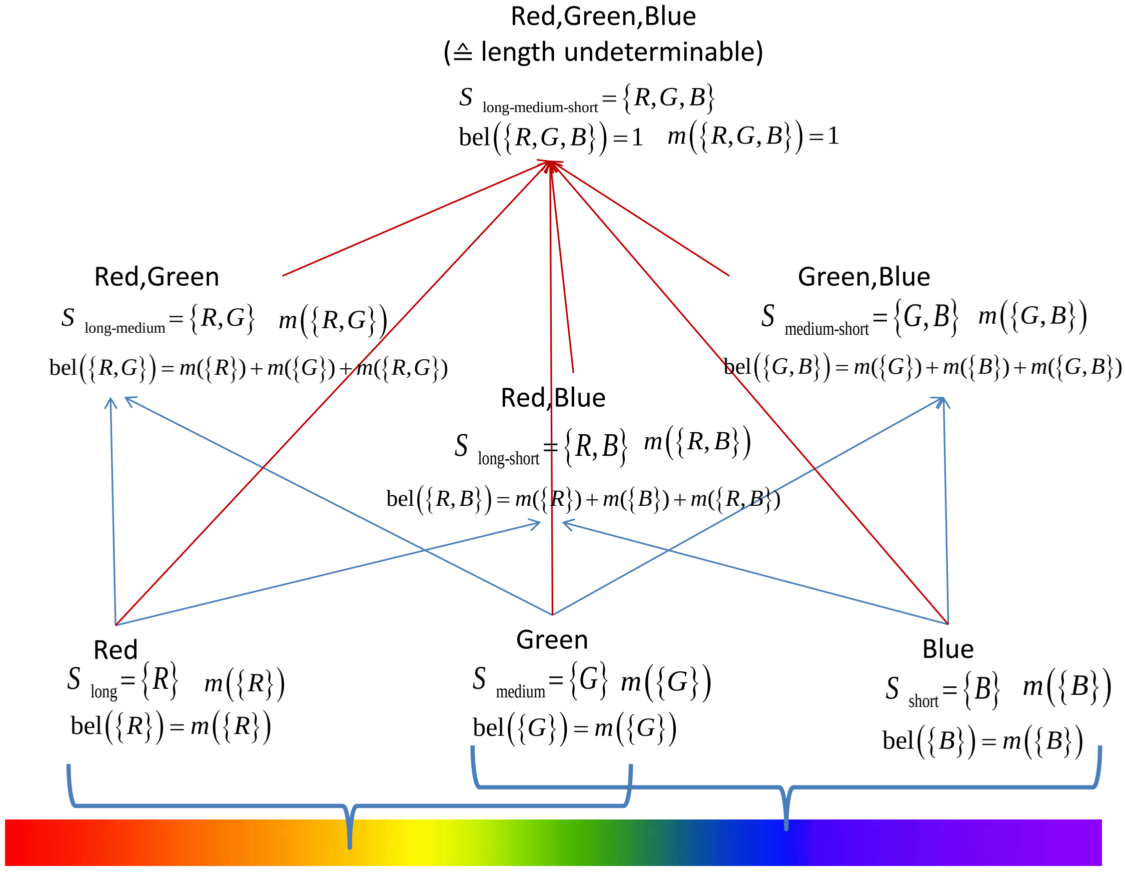

SuppFig. 1: Modelling subtotal resolution of measurement by a set of 3 events and subsets.
Illustration of a set of three possible events when detecting the color (wavelength) of light: $\Omega = \{A_1 = \text{red}, A_2 = \text{green}, A_3 = \text{blue}\}$, equivalent to wavelengths red≙long, green≙medium, blue≙short, see colorbar. The measurement device may yield a single element, e.g. 'blue', if it works at utmost resolution (bottom part of figure). However, an outcome might also be less specific, e.g. just 'long or medium', represented by $S_{\text{long-medium}} = \{\text{red, green}\}$ or 'medium or short' represented by $S_{\text{medium-short}} = \{\text{green, blue}\}$. General form of diagram: Hasse diagram, see p 263 of Weisstein[5].

In some cases a measurement will be less specific and yield an ambiguous result, e.g. that it was either event $A_1 \triangleq \text{red}$ or $A_2 \triangleq \text{green}$, see the example in SuppFig. 1. Likewise, an outcome may yield any other subset S of possible events, e.g. $S = \{A_1, A_3\}$ or $S = \{A_1, A_2, A_3\}$. To accommodate any possible type of information and ambiguity in outcome, one needs to consider all subsets of Ω, which is by definition the power set $\mathcal{P}(\Omega)$. As a starting point, DST assigns masses, $m(S)$, to each element of the power set. This is called 'basic belief assignment':

$$m(S): \mathcal{P}(\Omega) = 2^{\Omega} \to [0,1] \qquad \textbf{SuppEq. 1}$$

Specifying all masses fully specifies the evidence, including all uncertainties. In principle, masses may assume any values, provided the mass of the empty set is defined zero, all masses are positive and sum up to unity:

$$m(\varnothing) = 0 \quad \wedge \quad m(S) \geq 0 \quad \wedge \quad \sum_{S \in \mathcal{P}(\Omega)} m(S) = 1 \qquad \textbf{SuppEq. 2}$$

Apart from these formal limitations, the mass should be assigned to characterize the amount of belief strictly committed to the corresponding set, *S*, of outcomes.

The concept of *belief* bel(*S*) of a set *S* (of events) has been introduced qualitatively in the main part of this paper and can now be formalized by drawing on masses: Consider all members *R* of the power set whose elements also occur in *S*: $R \subseteq S$, and add up their masses:

$$\text{bel}(S) = \sum_{R \subseteq S} m(R) \qquad \textbf{SuppEq. 3}$$

This definition reflects that an outcome $S = \{A_1, .., A_j\}$ may arise from any (subset of) events contained in set *S*. It summarizes all reasons to believe in *S* with the available knowledge [6]. For a given input (sample) of light in the above example, a measuring device may be unable to discriminate medium from short wavelengths and yield nothing more than 'medium or short' as outcome (of low precision), $S = \{G, B\}$, see SuppFig. 1. Or else, for some other sample the device may by unable to discriminate long ($\{R\}$) from medium ($\{G\}$) and just yield $S = \{R, G\}$. When relating these outcomes to the underlying (exclusive) events, we obtain via SuppEq. 3:

$$\begin{aligned} \text{bel}(S = \{G, B\}) &= m(\{G\}) + m(\{B\}) + m(\{\text{G,B}\}) \\ \text{bel}(S = \{R, G\}) &= m(\{R\}) + m(\{G\}) + m(\{\text{R,G}\}) \end{aligned} \qquad \textbf{SuppEq. 4}$$

In addition to *belief*, DST also defines *plausibility*, pl(*S*), of a set *S*, . Descriptively, *plausibility* represents the amount of belief not strictly committed to the complement of S. For example, for $S = \{G\}$, the complement $\hat{S} = \Omega \setminus \{G\} = \{R, B\}$ and hence $\text{pl}(\{G\}) = 1 - \text{bel}(\{R, B\})$.

Formally, the plausibility of a subset S is defined by adding the masses $m(R)$ of all sets of events having non-empty intersections with S:

$$\mathrm{pl}(S) = \sum_{R \cap S \neq \varnothing} m(R) \quad \textbf{SuppEq. 5}$$

For the example shown in SuppFig. 1 we obtain the plausibility of $\{B\}$ by counting everything that contains (has non-empty intersection with) '*B*' (≙'blue'):

$$\mathrm{pl}(\{B\}) = m(\{B\}) + m(\{R,B\}) + m(\{G,B\}) + m(\{R,G,B\}) \quad \textbf{SuppEq. 6}$$

For a less precise outcome, e.g. $\{G,B\}$, the plausibility is given by:

$$\begin{aligned}\mathrm{pl}(\{G,B\}) &= m(\{G\}) + m(\{B\}) + \\ &\quad + m(\{G,B\}) + m(\{R,B\}) + \\ &\quad + m(\{R,B,G\}) \\ &= 1 - m(\{R\})\end{aligned} \quad \textbf{SuppEq. 7}$$

We note that plausibility always exceeds the belief in a given set *S*.

DST: General formalism and specification for hormone receptors

Single source of information (one receptor)

Specifically, for only two possible events, receptor positive versus negative, the above formalism boils down as follows: The set of events $\Omega_{\mathrm{Rez}} = \{\mathrm{Rez}^+, \mathrm{Rez}^-\}$, the power set $2^{\Omega_{\mathrm{Rez}}} = \{\{\mathrm{Rez}^+\}, \{\mathrm{Rez}^-\}, \{\mathrm{Rez}^+, \mathrm{Rez}^-\}, \varnothing\}$, and assigned masses are denoted by $m(\{\mathrm{Rez}^+\})$, $m(\{\mathrm{Rez}^-\})$ and $m(\{\mathrm{Rez}^+, \mathrm{Rez}^-\})$. We have:

$$\begin{aligned}\mathrm{bel}(\{\mathrm{Rez}^+\}) &= m(\{\mathrm{Rez}^+\}) = \alpha_{\mathrm{Rez}} \\ \mathrm{bel}(\{\mathrm{Rez}^-\}) &= m(\{\mathrm{Rez}^-\}) = \beta_{\mathrm{Rez}} \\ \mathrm{bel}(\{\mathrm{Rez}^+, \mathrm{Rez}^-\}) &= m(\{\mathrm{Rez}^+\}) + m(\{\mathrm{Rez}^-\}) + m(\{\mathrm{Rez}^+, \mathrm{Rez}^-\}) = \\ &= \alpha_{\mathrm{Rez}} + \beta_{\mathrm{Rez}} + \theta_{\mathrm{Rez}} = 1 \\ \mathrm{pl}(\{\mathrm{Rez}^+\}) &= m(\{\mathrm{Rez}^+\}) + m(\{\mathrm{Rez}^+, \mathrm{Rez}^-\}) = \alpha_{\mathrm{Rez}} + \theta_{\mathrm{Rez}} \\ \mathrm{pl}(\{\mathrm{Rez}^-\}) &= m(\{\mathrm{Rez}^-\}) + m(\{\mathrm{Rez}^+, \mathrm{Rez}^-\}) = \beta_{\mathrm{Rez}} + \theta_{\mathrm{Rez}}\end{aligned} \quad \textbf{SuppEq. 8}$$

For brevity of notation we use α, β and θ in the following.

Combining information from several sources with equal sets of outcomes (i.e. equal frames)

If several sources yield information about the same (physiological) item according to the same set of outcomes, $\Omega = \{A_1, A_2, ... A_n\}$), evidences need to be combined. One expects the result to depend neither on sequence nor on grouping of information, i.e. combination rules should be commutative and associative. This minimum requirement is fulfilled by several possible rules, out of which we introduce the Dempster '$\oplus_D$', and the Yager '$\oplus_Y$' addition rules. Due to associativity and commutativity, only pairwise addition needs to be specified: Given a member S of the power set, $S \in 2^{\Omega}$, and corresponding masses $m_1(S)$ and $m_2(S)$, referring to information source 1 and 2, respectively. According the Dempster ECR [6] masses are added follows:

$$m(S) = m_1(S) \oplus_D m_2(S) = \frac{\sum_{B \cap C = S} m_1(B) m_2(C)}{1 - \sum_{B \cap C = \varnothing} m_1(B) m_2(C)} \qquad \textbf{SuppEq. 9}$$

We note that the sum in the denominator, $\sum_{B \cap C = \varnothing} m_1(B) m_2(C)$, is a measure of conflict between the two sources of information. It acts as normalization, quantifies the amount of doubt and is no longer visible after combining information.

Applied to evidences in our frame, $\{\text{Rez}^+, \text{Rez}^-\}$, this boils down to Eq. 7 and Eq. 8 in the main text , representing fused information from gene and co-gene of a receptor.

Alternatively, the Yager ECR [7] yields (note the different cases $S \subset \Omega$ and $S = \Omega$):

$$\text{for } S \subset \Omega: \; m(S) = m_1(S) \oplus_Y m_2(S) = \sum_{B \cap C \subseteq S} m_1(B) m_2(C)$$

$$\text{for } S = \Omega: \; m(S) = m_1(S) \oplus_Y m_2(S) = m_1(S) \cdot m_2(S) + \sum_{B \cap C = \varnothing} m_1(B) m_2(C) \qquad \textbf{SuppEq. 10}$$

Note that we have applied the *Dempster* ECR to add evidences from genes and co-genes, since data of similar origin and widely concordant evidences have to be fused. In contrast, we used the *Yager* ECR to add evidence from IHC, since IHC is a different type of data source and occasionally in contradiction to gene expression

Combining information from different sources with different sets of outcomes (i.e. different frames)

Consider two sources of information, each yielding a specific set of outcomes (frame) $\Omega_1 = \{A_{11}, ... A_{1n_1}\}$ and $\Omega_2 = \{A_{21}, ... A_{2n_2}\}$. Combining information from these two sources yields possible outcomes represented in the product of sets of outcomes:

$$\Omega = \Omega_1 \times \Omega_2 \qquad \textbf{SuppEq. 11}$$

Now for each source of information, select a subset of outcomes which are of interest, $S_1 \subseteq \Omega_1$ and $S_2 \subseteq \Omega_2$. Combining these sources, the outcomes of interest are represented by $S = \{S_1, S_2\}$. Note that S is an element of the cross product set and its mass function $m(S)$ is given by an canonical product [7], $\otimes$:

$$m(S) = m_1(S_1) \otimes m_2(S_2)$$ **SuppEq. 12**

In order to illustrate the meaning of SuppEq. 12 formally within DST, one has to revert to masses defined on each of both the power sets: We recall that, for each receptor considered separately, the power set of events consists of 4 elements (including the empty set), e.g. for ER: $2^{\Omega_{ER}} = \{\{ER^+\},\{ER^-\},\{ER^+,ER^-\},\varnothing\}$. Note that sets with single elements represent crisp outcomes (singletons) whereas the element $\{ER^+,ER^-\}$ represents the indeterminable case. The elements of the ER power set appear as row-headings in SuppFig. 1. Likewise, elements of the PGR power set are column headings. To combine both receptors for building the 'hormone' evidence, we have to consider the product of these two power sets, $2^{\Omega_{ER}} \times 2^{\Omega_{PGR}}$, see SuppTable 1.

		power set of PGR outcomes			
		$\{PGR^+\}$ α_{PGR}	$\{PGR^-\}$ β_{PGR}	$\{PGR^+, PGR^-\}$ θ_{PGR}	$\varnothing$
power set of ER outcomes	$\{ER^+\}$ α_{ER}	$(\{ER^+\},\{PGR^+\})$ $\alpha_{ER}\alpha_{PGR}$	$(\{ER^+\},\{PGR^-\})$ $\alpha_{ER}\beta_{PGR}$	$(\{ER^+\},\{PGR^+,PGR^-\})$ $\alpha_{ER}\theta_{PGR}$	$\varnothing$
	$\{ER^-\}$ β_{ER}	$(\{ER^-\},\{PGR^+\})$ $\beta_{ER}\alpha_{PGR}$	$(\{ER^-\},\{PGR^-\})$ $\beta_{ER}\beta_{PGR}$	$(\{ER^-\},\{PGR^+,PGR^-\})$ $\beta_{ER}\theta_{PGR}$	$\varnothing$
	$\{ER^+, ER^-\}$ θ_{ER}	$(\{ER^+,ER^-\},\{PGR^+\})$ $\theta_{ER}\alpha_{PGR}$	$(\{ER^+,ER^-\},\{PGR^-\})$ $\theta_{ER}\beta_{PGR}$	$(\{ER^+,ER^-\},\{PGR^+,PGR^-\})$ $\theta_{ER}\theta_{PGR}$	$\varnothing$
	$\varnothing$	$\varnothing$	$\varnothing$	$\varnothing$	$\varnothing$

SuppTable 1: Product set of power sets for estrogen and progesterone.

Entries in yellow-shaded areas represent the power set of outcomes for ER and PGR, respectively. Entries in the blue shaded area represent the product set. Pairs of elements, making up the product set, are shown in parenthesis, e.g. $(\{ER^+\},\{PGR^+\})$. Below each pair the corresponding mass is given in terms of α, β and θ.

Masses of elements of the product set are computed as simple products of masses associated with column- and row-headings. Formally, given two sets of outcomes, $S_1 \in 2^{\Omega_1}$ and $S_2 \in 2^{\Omega_2}$, we have

$$\text{bel}((S_1,S_2)) = \sum_{\substack{A\subseteq S_1 \\ B\subseteq S_2}} m_1(A)\cdot m_2(B)$$ **SuppEq. 13**

This can also be understood according to conventional statistics as follows: The elements of power sets for ER and PGR are statistically independent outcomes and their masses may be related to probabilities. If so, any co-occurrence of outcomes, say $\{\mathrm{ER}^+\}$ and $\{\mathrm{PGR}^+\}$ occurs with the product of the respective probabilities.

As a final step we aggregate the result of utmost clinical relevance, the belief in being receptor positive. Clinically, the hormone status is considered positive (H^+) if at least one of both receptors is positive. Reading off from SuppTable 1, we note that ER is positive for all members of the first (horizontal) row, shaded in dark blue. All subsets within this row contribute to the belief, and their masses have to be summed up. Second we note that PGR is positive for all members of the first (vertical) column, also shaded in dark blue. These subsets also contribute with their masses to the sum. All in all we get for the belief in hormone receptor positivity, $\mathrm{bel}\left(\mathrm{H}^+\right)$:

$$\begin{aligned}\mathrm{bel}\left(\mathrm{H}^+\right) &= \alpha_{\mathrm{H}} = \alpha_{\mathrm{ER}}\alpha_{\mathrm{PGR}} + \alpha_{\mathrm{ER}}\beta_{\mathrm{PGR}} + \beta_{\mathrm{ER}}\alpha_{\mathrm{PGR}} + \alpha_{\mathrm{ER}}\theta_{\mathrm{PGR}} + \theta_{\mathrm{ER}}\alpha_{\mathrm{PGR}} = \\ &= \alpha_{\mathrm{ER}} + \alpha_{\mathrm{PGR}} - \alpha_{\mathrm{ER}}\alpha_{\mathrm{PGR}}\end{aligned}$$ **SuppEq. 14**

The last expression is the result already given in the main part of the paper.

To aggregate the belief in receptor negativity, neither of the receptors must be positive. Only a single element of the product set fulfills this requirement, shown in rose in SuppTable 1:

$$\mathrm{bel}\left(\mathrm{H}^-\right) = \beta_{\mathrm{H}} = \beta_{\mathrm{ER}}\beta_{\mathrm{PGR}}$$ **SuppEq. 15**

Together with the uncertainty

$$\theta_{\mathrm{H}} = 1 - \alpha_{\mathrm{H}} - \beta_{\mathrm{H}}$$ **SuppEq. 16**

we may enter the clinical decision criterion, eq. 11 from the main text, whether or not hormone therapy should be applied.

To complete, plausibilities can readily be deduced:

$$\begin{aligned}\mathrm{pl}\left(\mathrm{H}^+\right) &= 1 - \beta_{\mathrm{H}} \\ \mathrm{pl}\left(\mathrm{H}^-\right) &= 1 - \alpha_{\mathrm{H}}\end{aligned}$$ **SuppEq. 17**

Concordance between IHC and gene expression

SuppTable 2: Studywise Concordance between IHC and gene expression. Receptor status given by IHC was reevaluated by the ODDS-method[8] with a crisp cutoff, i.e. without a range for uncertain samples. Analysis was performed separately for ER and PGR. For comparison, ER estimates via scmgene from genefu[9] are given (scmgene does not give PGR estimates).

N_{IHC} number of available IHC measurements for ER and PGR, respectively. $\Delta_{IHC,ODDS}$ and $\Delta_{IHC,scmgene}$: number of discordant samples regarding ER. Not that 3 studies neither had IHC-measurement nor could they be imputed safely. $\Delta_{IHC,scmgene}$: samples discordant between IHC and scmgene of genefu.

	ER			PGR	
**Study**	N_{IHC}	$\Delta_{IHC,ODDS}$	$\Delta_{IHC,scmgene}$	N_{IHC}	$\Delta_{IHC,ODDS}$
GSE5460	17	0	0	0	0
GSE6532	78	0	0	77	16
GSE12276	0	0	0	0	0
GSE12777	0	0	0	0	0
GSE16391	50	1	1	50	12
GSE16446	84	0	0	0	0
GSE18728	15	3	3	14	6
GSE18864	60	0	0	60	3
GSE19615	79	2	2	79	10
GSE20685	0	0	0	0	0
GSE20711	52	9	0	0	0
GSE22035	27	0	0	0	0
GSE23177	80	2	0	0	0
GSE26639	144	11	14	142	18
GSE27120	26	6	2	26	5
GSE29431	23	3	4	23	2
GSE31448	286	21	37	271	32
GSE36771	86	6	6	85	7
GSE42568	63	15	0	0	0
GSE43358	43	3	3	43	1
GSE43365	98	3	2	98	4
GSE46222	26	6	0	0	0
GSE47389	47	1	2	47	1

GSE48390	34	2	0	0	0
GSE48905	20	1	0	0	0
GSE50567	25	0	0	0	0
GSE58792	33	1	0	0	0
GSE58812	107	12	18	107	3
GSE61304	38	0	1	33	4
GSE65194	81	0	3	55	0
GSE71258	77	11	11	77	15
GSE76124	198	30	49	198	29
GSE76274	44	1	0	44	5
GSE87007	24	1	0	24	2
GSE88770	108	8	7	107	19
GSE95700	54	0	3	54	1
∑	2227	159	168	1714	195

It is interesting to evaluate if discordant cases fall into a few of the 38 studies or if they are evenly distributed. For an evaluation, we consider a given study, with N_{IHC} samples and an observed discordance rate $p = \Delta_{IHC\text{-}ODDS} / N_{IHC}$. For shortness of notation, we put $\Delta_{IHC\text{-}ODDS} = k$ and $N_{IHC} = n$. For this study, a confidence interval for the discordance rate is constructed as follows:

Since discordance occurs with probability p, the probability to find k discordant cases out of n samples is given by the binomial distribution

$$\Pr(k \mid n, p) = \binom{n}{k} p^k \left(1-p\right)^{n-k} \qquad \textbf{SuppEq. 18}$$

To derive the confidence interval for p, Bayes' theorem can be applied

$$\Pr(p \mid n, k) = \Pr(k \mid n, p) \cdot \Pr\left(p\right) \qquad \textbf{SuppEq. 19}$$

Since for an individual study no a priori information about p is available, we may assume that each value of $p \in \left[0,1\right]$ is equally probable (uniform distribution):

$$\Pr(p) = I_{[0,1]} \qquad \textbf{SuppEq. 20}$$

For normalization

$$\int_0^1 \Pr(p \mid n, k) dp = 1 \qquad \textbf{SuppEq. 21}$$

the appropriate factor is

$$\Pr(p \mid n,k) = \frac{\Gamma(k+1)\cdot\Gamma(n+k+1)}{\Gamma(n+2)} p^k (1-p)^{n-k}$$ **SuppEq. 22**

This turns out to exactly equal the Beta-distribution

$$\text{betapdf}(p \mid k+1, n-k+1) = \frac{1}{B(k+1, n-k+1)} p^k (1-p)^{n-k}$$ **SuppEq. 23**

From this, confidence intervals can be derived as usual and are shown in SuppFig. 2 and figure 3 in the main text.

If a confidence interval overlaps with the mean discordance rate over all 38 studies ($p = 159 / 2227 \sim 0.071$, dotted line), the study in question is considered 'normal'. Should the lower confidence limit exceed this mean, the study is considered 'above' average discordance (red). If the upper confidence limit is below this mean, the study is considered 'below' average discordance (green), otherwise 'normal' (blue).

The expectation value of the Beta-distribution is given by

$$E(p \mid k+1, n-k+1) = \frac{k+1}{n+2}$$ **SuppEq. 24**

and is shown as x-marker in the figure. Note that for finite n this expectation value is slightly below the 'naïve' and unbiased ratio k / n but has the following advantage: While the unbiased estimate k / n describes data already obtained, the expectation value of the beta-distribution ($k + 1 / n + 2$) reflects what is to be expected from coming measurements under the very same conditions of the study considered. Asymptotically, after all

$$\frac{k+1}{n+2} \underset{n\to\infty}{\sim} \frac{k}{n}$$ **SuppEq. 25**

the estimate converges to unbiased.

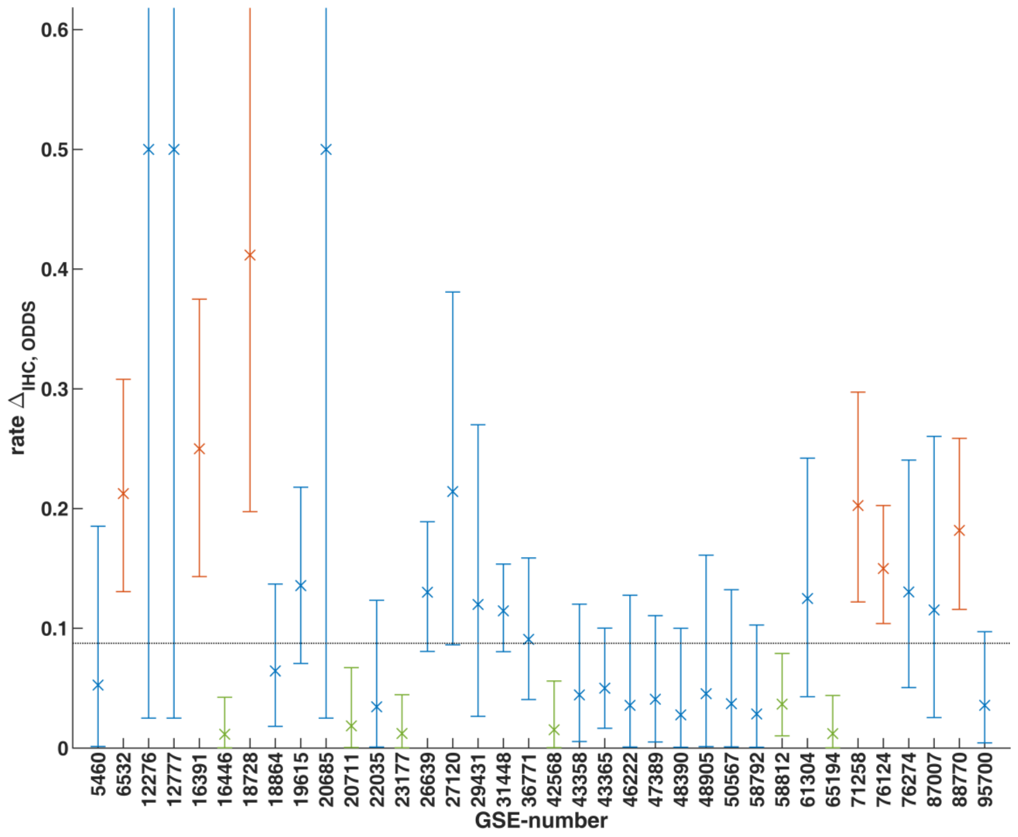

SuppFig. 2: **Control chart for progesterone receptor status: differences between IHC and ODDS gene expression estimates**
x-axis: label of study. Y-axis: rate of difference. Dotted line: overall mean rate of differences between IHC and ODDS, $p = 195/1714 = 0.11$, see SuppTable 2. Marker (x) within error bar: expectation value of rate of difference. Lower and upper bounds of error bars denote 0.025 and 0.975 confidence limits according to the corresponding beta-distribution. If an upper bound lies below the dotted line, the respective study has a discrepancy rate significantly below average (green). If a lower bound lies above the dotted line, the respective study has a discrepancy rate significantly above average (red).

.

Receptor Status and Survival

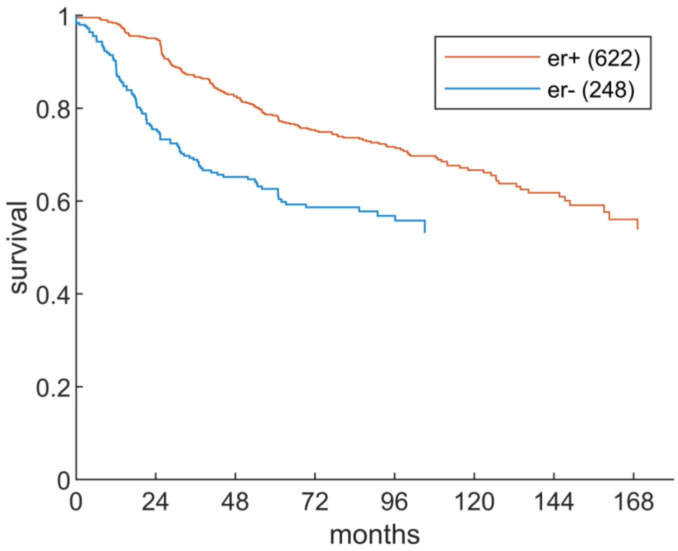

SuppFig. 3: **Survival free from recurrence for estrogen**
Kaplan Meier estimates of survival free from recurrence for 870 patients (who had rfs-data) for different estrogen receptor status. Log-rank test *p* = 8.1e-07.

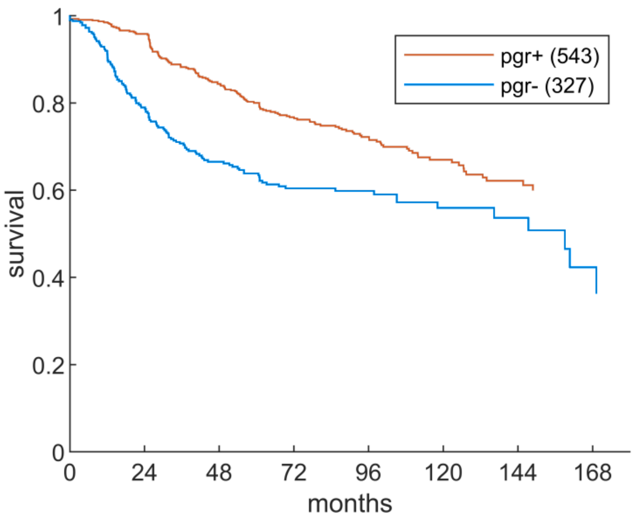

SuppFig. 4: **Survival free from recurrence for progesterone**
Kaplan Meier estimates of survival free from recurrence for 870 patients (who had rfs-data) for different progesterone receptor status. Log-rank test *p* = 5.2e-07.

References in Supplementary Material

1 Yang, J. B. & Xu, D. L. Evidential reasoning rule for evidence combination. *Artif. Intell.* **205**, 1-29 (2013).

2 Liu, L. & Yager, R. R. in *Classic Works of the Dempster-Shafer Theory of Belief Functions* (eds Roland R. Yager & Liping Liu) 1-34 (Springer Berlin Heidelberg, 2008).

3 Beynon, M., Curry, B. & Morgan, P. The Dempster–Shafer theory of evidence: an alternative approach to multicriteria decision modelling. *Omega* **28**, 37-50 (2000).

4 Jøsang, A. & Pope, S. Dempster´s rule as seen by little colored balls. *Computational Intelligence* **28**, 453-474 (2012).

5 Weisstein, E. W. *CRC Concise Encyclopedia of Mathematics*. (CRC Press, 2002).

6 Fontani, M., Bianchi, T., De Rosa, A., Piva, A. & Barni, M. A Framework for Decision Fusion in Image Forensics Based on Dempster–Shafer Theory of Evidence. *IEEE Transactions on Information Forensics and Security* **8**, 593-607, doi:10.1109/TIFS.2013.2248727 (2013).

7 Yager, R. R. On the dempster-shafer framework and new combination rules. *Information Sciences* **41**, 93-137, doi:https://doi.org/10.1016/0020-0255(87)90007-7 (1987).

8 Kenn, M. *et al.* Co-expressed genes enhance precision of receptor status identification in breast cancer patients. *Breast Cancer Res. Treat.* **172**, 313-326, doi:10.1007/s10549-018-4920-x (2018).

9 Gendoo, D. M. *et al.* Genefu: an R/Bioconductor package for computation of gene expression-based signatures in breast cancer. *Bioinformatics* **32**, 1097-1099 (2016).
